# Epidemiology of Carbapenemase-Producing *Klebsiella pneumoniae* in a Hospital, Portugal

**DOI:** 10.3201/eid2509.190656

**Published:** 2019-09

**Authors:** Marta Aires-de-Sousa, José Manuel Ortiz de la Rosa, Maria Luísa Gonçalves, Ana Luísa Pereira, Patrice Nordmann, Laurent Poirel

**Affiliations:** Escola Superior de Saúde da Cruz Vermelha Portuguesa, Lisbon, Portugal (M. Aires-de-Sousa);; Instituto de Tecnologia Química e Biológica António Xavier, Universidade Nova de Lisboa, Oeiras, Portugal (M. Aires-de-Sousa);; Université de Fribourg, Fribourg, Switzerland (M. Aires-de-Sousa, J.M. Ortiz de la Rosa, P. Nordmann, L. Poirel); Hospital SAMS, Lisbon (M.L. Gonçalves, A.L. Pereira);; Swiss National Reference Center for Emerging Antibiotic Resistance, Fribourg (P. Nordmann, L. Poirel);; University of Lausanne, Lausanne, Switzerland (P. Nordmann);; University Hospital Centre, Lausanne (P. Nordmann)

**Keywords:** carbapenemase, Klebsiella pneumoniae, Portugal, KPC-3, OXA-181, GES-5, BEL-1, bacterial infections, antimicrobial resistance, nosocomial infections

## Abstract

We aimed to provide updated epidemiologic data on carbapenem-resistant *Klebsiella pneumoniae* in Portugal by characterizing all isolates (N = 46) recovered during 2013–2018 in a 123-bed hospital in Lisbon. We identified *bla*_KPC-3_ (n = 36), *bla*_OXA-181_ (n = 9), and *bla*_GES-5_ (n = 8) carbapenemase genes and observed co-occurrence of *bla*_KPC-3_ and *bla*_GES-5_ in 7 isolates. A single GES-5–producing isolate co-produced the extended-spectrum β-lactamase BEL-1; both corresponding genes were co-located on the same ColE1-like plasmid. The *bla*_OXA-181_ gene was always located on an IncX3 plasmid, whereas *bla*_KPC-3_ was carried on IncN, IncFII, IncFIB, and IncFIIA plasmid types. The 46 isolates were distributed into 13 pulsotypes and 9 sequence types. All isolates remained susceptible to ceftazidime/avibactam, but some exhibited reduced antimicrobial susceptibility (MIC = 3 mg/L).

*Klebsiella pneumoniae* is a major cause of hospital-acquired infections, mainly responsible for urinary, respiratory, and bloodstream infections, as well as infections in intensive care unit (ICU) patients. The emergence of antimicrobial resistance, in particular the rise of carbapenem-resistant isolates, is a serious concern for the management of infections caused by *K. pneumoniae* because treatment alternatives are limited. Therefore, carbapenem-resistant *K. pneumoniae* are ranked among the recently published World Health Organization list of antibiotic-resistant “priority/critical” pathogens, for which research and development of new antibiotics is required (*1*).

Carbapenem resistance in *K. pneumoniae* arises from 2 main mechanisms: permeability defects combined with overexpression of a β-lactamase with weak carbapenemase activity (mostly CTX-M or AmpC cephalosporinases) and the acquisition of carbapenemases (*2*). Carbapenemase enzymes belong to 3 different Ambler classes (*3*). Class A includes the KPC-type enzyme, which has been extensively reported in *K. pneumoniae*; to date, although >20 different KPC variants have been described, KPC-2 and KPC-3 remain the most common types (*4*). In addition, a few studies reported the carbapenemase GES-5 (a point mutant derivative of the extended-spectrum β-lactamase [ESBL] GES-1) in *K. pneumoniae* (*5*). Class B includes the metallo-β-lactamases, which are mainly NDM-, VIM-, and IMP-type enzymes, and class D includes OXA-48–like β-lactamases.

The first clinical carbapenemase-producing *K. pneumoniae* in Portugal was isolated at a Lisbon hospital in 2009 (*6*). Since then, only sporadic infection isolates and single hospital cases have been reported (*7*,*8*), as well as a single outbreak of KPC-3–producing *K. pneumoniae* in 2013 (*9*). Surprisingly, no other carbapenemase type, such as OXA-48 and derivatives that are widespread in other countries in Europe (*8*), has been identified in *K. pneumoniae* in Portugal so far. A survey of *Enterobacteriaceae* collected in 13 hospitals in Portugal confirmed a low prevalence of carbapenemase producers in the country until 2013 (35/2105 [1.7%]) and a predominance of *K. pneumoniae* KPC-3 producers (*10*). However, in 2017, according to the annual report of the European Centre for Disease Prevention and Control on antimicrobial resistance in Europe, 8.6% of *K. pneumoniae* causing invasive infections in Portugal were resistant to carbapenems (*11*). That report also showed that Portugal faced an annual increasing trend of carbapenem resistance among *K. pneumoniae* since 2014 (1.8% in 2014, 3.4% in 2015, 5.2% in 2016, and 8.6% in 2017), exceeding the overall prevalence for Europe (7.2%). Nevertheless, data on the molecular epidemiology of nosocomial carbapenemase-producing *K. pneumoniae* in Portugal are still limited, and the existing studies include isolates recovered until 2014 only (*10*,*12*,*13*). Therefore, the aim of our study was to provide updated epidemiologic data on contemporary carbapenemase-producing *K. pneumoniae* in an acute-care facility hospital in Portugal by characterizing a collection of nonrepetitive isolates recovered during a 6-year period (2013–2018).

## Materials and Methods

### Bacterial Isolates

Carbapenemase-producing *K. pneumoniae* isolates (N = 46) recovered during 2013 (n = 1), 2014 (n = 1), 2016 (n = 9), 2017 (n = 12), and 2018 (n = 23) in a 123-bed hospital (SAMS Hospital) in Lisbon, Portugal, were used for our study. All isolates were from single patients and recovered from colonization and infection sites: rectal swabs (n = 20), urine (n = 15), sputum (n = 4), blood (n = 4), and other sites (n = 3). The isolates were obtained from 22 inpatients (ICU, n = 7; medicine ward, n = 11; surgery ward, n = 3; other, n = 1) and 24 outpatients (emergency department, n = 21; ambulatory consultation, n = 3).

### Susceptibility Testing

We performed antimicrobial susceptibility testing by using the disk diffusion method on Mueller-Hinton agar plates (Bio-Rad, http://www.bio-rad.com) for amoxicillin, amoxicillin/clavulanic acid, ticarcillin, ticarcillin/clavulanic acid, piperacillin, piperacillin/tazobactam, temocillin, ceftazidime, cefotaxime, cefoxitin, aztreonam, imipenem, ertapenem, meropenem, gentamicin, amikacin, ciprofloxacin, tigecycline, trimethoprim-sulfamethoxazole (SXT), and fosfomycin, following EUCAST recommendations (http://www.eucast.org). We determined MICs for imipenem, ertapenem, meropenem, and ceftazidime/avibactam by Etest (bioMérieux, http://www.biomerieux.com). Interpretation of MICs and zone diameters followed EUCAST breakpoint tables (http://www.eucast.org/fileadmin/src/media/PDFs/EUCAST_files/Breakpoint_tables/v_9.0_Breakpoint_Tables.pdf).

We assessed selection of carbapenemase producers by using the Rapidec Carba NP test (bioMérieux) (*14*). In addition, we evaluated colistin susceptibility by using the Rapid Polymyxin NP test (ELITechGroup Microbiology, http://www.elitechgroup.com) (*15*).

### Molecular Analysis

We identified carbapenemases (*16*) and ESBL genes (*17*) and detected the *mcr*-type colistin-resistance gene (*18*) by using PCR amplification, followed by sequencing of the amplicons. We used standard PCR conditions to amplify the β-lactamase gene *bla*_CMY_, encoding plasmid-mediated cephalosporinases (*19*), and the *qnr*S quinolone resistance gene (*20*). We detected the *bla*_OXA-9_ gene encoding a narrow-spectrum class D β-lactamase by using PCR amplification with primers OXA-9 FW 5′-ATGAAGGATACCTTGATGAAAAA-3′ and OXA-9 RW 5′-TCATTTGTTACCCATCAACACG-3′.

We evaluated the clonal relationship of the isolates by pulsed-field gel electrophoresis (PFGE), as described previously (*21*). We performed multilocus sequence typing (MLST) for a representative strain of each PFGE type and assigned sequence types (STs) by using the MLST database for *K. pneumoniae* (http://bigsdb.pasteur.fr/klebsiella/klebsiella.html).

### Conjugation Experiments and Plasmid Analysis

We performed mating-out assays by using the azide-resistant *Escherichia coli* J53 as the recipient. We separately inoculated donors carrying *E. coli* J53 and *bla*_KPC-3_ or *bla*_OXA-181_ overnight into LB broth (5 mL) and incubated, then subsequently mixed samples at a ratio of 10:1 (donor:recipient) for 5 h. We deposited 100 µL of this mix onto 22-µm filters and incubated overnight at 37°C on LB agar plates. After the incubation, we resuspended filters in 0.85% NaCl, then plated 100 µL of this mixture onto LB agar plates supplemented with ticarcillin (100 µg/mL) and azide (100 µg/mL). We performed susceptibility testing for all *E. coli* transconjugants and assessed positivity for *bla*_KPC-3_ or *bla*_OXA-181_ by using PCR.

We classified plasmids according to their incompatibility group by using the PCR-based replicon typing method as described previously (*22*). We characterized the plasmid carrying *bla*_BEL-1_ and *bla*_GES-5_ genes by using primers for ColE1-like plasmids, as previously described (*23*).

## Results

Among the 46 carbapenemase-producing isolates, the most common carbapenemase identified was KPC-3 (n = 36 [78%]), followed by OXA-181 (n = 9 [20%]) and GES-5 (n = 8 [17%]) (Table). Seven isolates co-harbored >1 carbapenemase gene (*bla*_KPC-3_ and *bla*_GES-5_). The *bla*_OXA-9_ gene encoding a narrow-spectrum class D β-lactamase was identified in 31 isolates, all of which were KPC-3 producers. A single GES-5–producing isolate co-produced the ESBL BEL-1.

**Table:** Characteristics of 46 carbapenemase-producing *Klebsiella pneumoniae* isolates collected in a hospital in Portugal, 2013–2018*

PFGE type	ST	Isolation year†	No. isolates	Resistance determinants‡	Plasmid type§	MIC for ceftazidime/ avibactam, mg/L	Nonsusceptible phenotype¶
A	147	**2016** (6)	7 (15%)	***bla*_KPC-3_**, ***bla*_GES-5_**, *bla*_OXA-9_, *qnrS*	IncFII/ColE1	1.5–3	PPT, TCC, AMC, CZD, CTX, FOX, ATM, IMP, ETP, MEM, GMI (6), AKN (6), CIP, TIG (1), SXT
		2018 (1)			IncN/ColE1		
B	147	2016 (2)	6 (13%)	***bla*_KPC-3_,** *bla*_OXA-9_, *qnrS*	IncFII	1–2	PPT, TCC, AMC, CZD, CTX, FOX, ATM, IMP, ETP, MEM, GMI, AKN, CIP, TIG, SXT
		2017 (2)					
		2018 (2)					
C	231	2014 (1)	3	***bla*_KPC-3_,** *qnrS*	IncFII	1.5	PPT, TCC, AMC, CZD, CTX, FOX, ATM, IMP (2), ETP, MEM, GMI (2), AKN (2), CIP, TIG (1), SXT
		2017 (1)		***bla*_KPC-3_,** *bla*_OXA-9_, *qnrS*			
		2018 (1)		***bla*_KPC-3_,** *bla*_OXA-9_, *qnrS*			
D	13	2017 (2)	7 (15%)	***bla*_KPC-3_,** *bla*_OXA-9_, *qnrS*	IncFIB	0.75–2	PPT, TCC, AMC, CZD, CTX, FOX, ATM, IMP (4), ETP, MEM, GMI (5), AKN (4), CIP (2), TIG (1), FOS (1), SXT
		**2018** (5)		***bla*_KPC-3_,** *bla*_OXA-9_ (3), *qnrS*			
E	147	2017 (2)	3	***bla*_KPC-3_,** *bla*_OXA-9_ (1), *qnrS*	IncFIIA	0.75–1.5	PPT, TCC, AMC, CZD, CTX, FOX, ATM, IMP, ETP, MEM, GMI (1), AKN (1), CIP, TIG (1), SXT (2)
		2018 (1)		***bla*_KPC-3_,** *bla*_OXA-9_, *qnrS*			
F	960	2013	1	***bla*_KPC-3_,** *qnrS*	IncN	2	PPT, TCC, AMC, CZD, CTX, FOX, ATM, IMP, ETP, MEM, SXT
G	348	2017 (1)	7 (15%)	***bla*_KPC-3_,** *bla*_OXA-9_, *qnrS*	IncFII	0.5–2	PPT, TCC, AMC, CZD, CTX, FOX, ATM, IMP (6), ETP, MEM, GMI (6), AKN (5), CIP (6), TIG (2), FOS (1), SXT (6), COL (1)
		**2018** (6)		***bla*_KPC-3_,** *bla*_OXA-9_ (5), *qnrS*			
H	45	2017	1	***bla*_KPC-3_,** *bla*_OXA-9_, *qnrS*	IncFII	0.75	PPT, TCC, AMC, CZD, CTX, FOX, ATM, IMP, ETP, CIP, SXT
I	35	2018	1	***bla*_KPC-3_,** *bla*_OXA-9_, *qnrS*	IncN	0.75	PPT, TCC, AMC, CZD, CTX, FOX, ATM, IMP, ETP, MEM, GMI, AKN, CIP, SXT
J	17	2016 (1)	6 (13%)	***bla*_OXA-181_,** *qnrS*	IncX3	0.125–0.25	PPT, TCC, AMC, CZD, CTX, FOX (5), ATM, IMP (3), ETP, GMI (5), AKN (3), CIP, SXT
		2017 (1)					
		**2018** (4)					
L	17	2017	1	***bla*_OXA-181_,** *qnrS*	IncX3	0.25	PPT, TCC, AMC, CZD, CTX, ATM, ETP, GMI, CIP, SXT
M	35	2017 (1)	2	***bla*_OXA-181_,** *qnrS*	IncX3	0.25	PPT, TCC, AMC, CZD, CTX, FOX, ATM, ETP, GMI, CIP, SXT
		2018 (1)					PPT, TCC, AMC, CZD, CTX, ATM, ETP, CIP, SXT
N	29	2018	1	***bla*_GES-5_,** *bla*_BEL-1_, *qnrS*	ColE	0.5	PPT, TCC, AMC, CZD, FOX, FOS

*AKN, amikacin; AMC, amoxicillin/clavulanic acid; ATM, aztreonam; CIP, ciprofloxacin; COL, colistin; CTX, cefotaxime; CZD, ceftazidime; ETP, ertapenem; FOS, fosfomycin; FOX, cefoxitin; GMI, gentamicin; IMP, imipenem; MEM, meropenem; PFGE, pulsed-field gel electrophoresis; PPT, piperacillin/tazobactam; ST, sequence type (determined by multilocus sequence typing); SXT, trimethoprim/sulfamethoxazole; TCC, ticarcillin/clavulanic acid; TIG, tigecycline. †Periods for which the clonal type is clearly predominant shown in bold. Numbers in parentheses indicate number of isolates recovered for each year listed. ‡Carbapenemase genes are shown in bold. Numbers in parentheses indicate the number of isolates that carry the resistance gene, if not all. §Type of the plasmid carrying the carbapenemase gene. ¶Numbers in parentheses indicate number of isolates that are nonsusceptible to the antimicrobial agent, if not all.

Mating-out assays followed by PCR-based replicon typing revealed that the *bla*_OXA-181_ gene was always located onto an IncX3 plasmid, whereas the *bla*_KPC-3_ gene was carried onto IncN, IncFII, IncFIB, and IncFIIA plasmid types. The genes *bla*_GES-5_ and *bla*_BEL-1_ were co-located on the same ColE1-like plasmid.

Antimicrobial susceptibility testing showed that 13 (28%) of the 46 carbapenemase producers were susceptible to imipenem (MIC <0.5 mg/L) and 10 (22%) isolates were susceptible to meropenem (MIC <1 mg/L). Eight (17%) isolates were susceptible to both carbapenems, and all except 1 were OXA-181 producers (p<0.001). The remaining isolate harbored *bla*_GES-5_ and *bla*_BEL-1_ and was also susceptible to ertapenem (MIC 0.25 mg/L). This isolate was the only isolate showing susceptibility to aztreonam. Despite the low MICs for carbapenems, all isolates grew on the chromID CARBA SMART agar (bioMérieux); the OXA-181 producers grew on the OXA side, whereas the GES-5/BEL-1 producer grew on the CARBA side, as did all KPC producers.

A substantial proportion of isolates showed reduced susceptibility to SXT (93%), ciprofloxacin (85%), gentamicin (76%), and amikacin (63%). In addition, nonsusceptibility to tigecycline was found in 28% of the isolates, resistance to fosfomycin in 3 isolates, and resistance to colistin in 1 isolate (i.e., negative for *mcr*-type genes). All isolates remained susceptible to ceftazidime/avibactam (MIC values ranging from 0.125 to 3 mg/L), although some exhibited reduced susceptibility (resistance breakpoint >8 mg/L). Isolates co-possessing *bla*_KPC-3_ and *bla*_GES-5_ genes showed higher MICs for ceftazidime/avibactam (>1.5 mg/L [p = 0.014]), whereas isolates producing carbapenemase OXA-181 had lower values (<0.25 mg/L [p<0.001]).

The plasmid-mediated quinolone resistance gene *qnrS* was present in all isolates. No isolates carried the β-lactamase gene *bla*_CMY_.

PFGE analysis distributed the 46 isolates into 13 pulsotypes (Table). PFGE type A included the 7 isolates co-producing KPC-3 and GES-5 carbapenemases. The 29 isolates producing KPC-3 alone belonged to 8 different PFGE types: B (n = 6), C (n = 3), D (n = 7), E (n = 3), F (n = 1), G (n = 7), H (n = 1), and I (n = 1). PFGE types J (n = 6), L (n = 1), and M (n = 2) included the 9 isolates that were positive for *bla*_OXA-181_, and the 1 isolate co-producing GES-5 and BEL-1 belonged to PFGE type N.

By using MLST, we found that the 13 PFGE types corresponded to 9 STs (Table). These STs were ST147 (n = 16 [35%]), ST13 (n = 7 [15%]), ST17 (n = 7 [15%]), ST348 (n = 7 [15%]), ST231 (n = 3 [7%]), ST35 (n = 3 [7%]), ST29 (n = 1), ST45 (n = 1), and ST960 (n = 1).

Analyzing the different clonal types over time (Figure), we observed that clone F-ST960 (KPC-3), recovered in 2013 in a single isolate, was no longer detected in the following years. Clone A-ST147 (KPC-3/GES-5), which was 1 of the 2 major clones found in our study, was clearly predominant in 2016 (6/7 isolates) but became a sporadic clone in 2018 (1 isolate). Conversely, clone G-ST348 (KPC-3), which emerged as a single isolate in 2017, became the major clone in 2018; this clone and clone D-ST13 are currently the predominant KPC-3 clones in the hospital. The first OXA-181 isolate was detected in 2016 (clone J-ST17), and although the 9 isolates producing this enzyme were distributed into 3 clones, J-ST17 became the predominant OXA-181 clone in 2018. The diversity of clones among carbapenemase producers increased over time together with the increase of isolates, especially in 2017 (9 clones among 12 isolates) (Figure).

**Figure:**
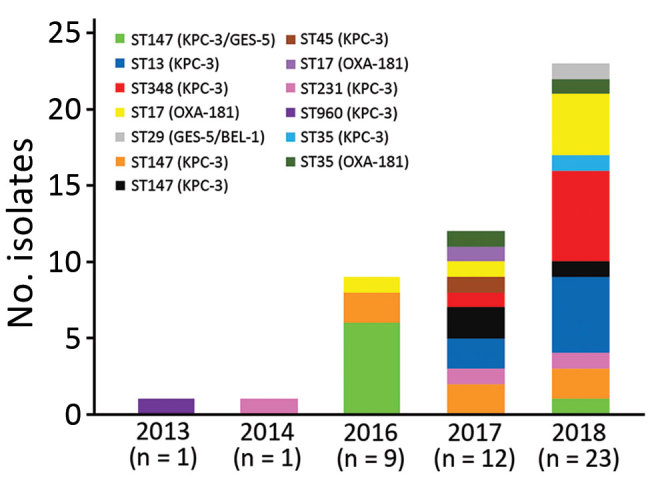
Evolution of clonal types of carbapenem-producing *Klebsiella pneumoniae* in a hospital in Portugal, 2013–2018. ST, sequence type.

## Discussion

Our epidemiologic study describes the evolution of carbapenem-resistant *K. pneumoniae* over time in a hospital in Portugal, including contemporary isolates recovered after 2014. We report an increasing rate of carbapenemase producers in this hospital since 2016, which is consistent with the rise of carbapenem-resistant isolates in the country as reported by the European Centre for Disease Prevention and Control (*11*).

We found a clear predominance (78%) of *K. pneumoniae* KPC-3 producers, as observed in previous studies describing isolates in Portugal until 2014 (*10*,*24*,*25*). The KPC-3 producers belonged to a high diversity of clones (n = 9), and the *bla*_KPC-3_ gene was carried onto 4 plasmid types (IncN, IncFII, IncFIB, and IncFIIA). Therefore, the increasing number of KPC-3–producing isolates in this hospital might result mainly from recurrent introductions of those strains rather than the dissemination of specific plasmids or particular clones. ST147 was the predominant background type identified, either producing KPC-3 or OXA-181, which correlates with previous findings showing that this ST (belonging to clonal complex 258, as commonly found with KPC-2 or KPC-3 producers worldwide) was mainly associated with the spread of KPC-3-producing *K. pneumoniae* in the community in North Portugal (*25*).

Furthermore, most KPC-3–producing isolates (n = 31 [86%]) also carried the narrow-spectrum class D β-lactamase *bla*_OXA-9_. This association has been previously described in an *Enterobacter cloacae* strain from a patient in France who was transferred from a hospital in the United States (*26*) and also among *K. pneumoniae* isolates from Italy (*27*). The co-production of OXA-9 contributes to the high-level resistance observed for amoxicillin/clavulanic acid because KPC-3 activity remains partially antagonized by clavulanate.

We have shown that OXA-181 producers have emerged among clinical isolates in Portugal and have been appearing at an increasing rate over time in this hospital. This carbapenemase, which is emerging worldwide, was found in isolates recovered since 2016, and its prevalence among carbapenemases is on the rise, which is a worrying and unexplained phenomenon. We recently described the massive occurrence of *K. pneumoniae* isolates carrying the *bla*_OXA-181_ gene located onto an IncX3 plasmid in Angola (*21*,*28*) and also some isolates in São Tomé and Príncipe (*29*). The genetic backgrounds of those OXA-181–producing *K. pneumoniae* were different, with no ST17 detected, in contrast to our findings in this study; nevertheless, the identification of a very similar plasmid carrying *bla*_OXA-181_ likely suggests a wide plasmid dissemination as a source of carbapenemase gene acquisition. The frequent exchange of populations between these 2 countries, which are former colonies of Portugal, and Portugal itself could have driven the intercontinental spread of carbapenemase producers. Some recent studies have underscored the emergence of OXA-181 carbapenemase mediated by acquisition of similar IncX3-type plasmids, such as in China or South Africa (*30*,*31*), suggesting that spread of this carbapenem-resistance trait could be linked in large part to a wide plasmid diffusion.

Our data indicate the emergence and spread of *K. pneumoniae* isolates co-producing KPC-3 and GES-5. GES-5 has been found previously in Portugal in a *K. pneumoniae* environmental isolate (*32*) and in a single clinical strain (*24*) but never in combination with KPC. The fact that isolates co-possessing *bla*_KPC-3_ and *bla*_GES-5_ showed higher MICs to ceftazidime/avibactam is of concern.

β-lactamase GES-5 has been reported in *K. pneumoniae*, mainly among sporadic isolates with origins in Asia, namely from a patient from France who had been hospitalized in Bangkok, Thailand (*33*), in 1 isolate from China (*34*), and in 1 isolate from South Korea (*35*). A single study reported the spread of *K. pneumoniae* GES-5–producing isolates recovered during 2012–2013 in South Africa that carried *bla*_GES-5_ in an IncQ plasmid (*36*).

Our study identified 1 isolate co-producing GES-5 and the ESBL BEL-1. The 2 corresponding genes were embedded in the same class 1 integron structure, as first and second gene cassette positions, respectively (data not shown). The occurrence of the *bla*_BEL-1_ gene in an enterobacterial isolate is noteworthy because this gene has been identified mainly in *Pseudomonas aeruginosa*. Likewise, *bla*_GES-5_ is commonly identified in *P. aeruginosa*. Therefore, our finding suggests that this ColE1 plasmid bearing these 2 uncommon broad-spectrum β-lactamases might originate from *P. aeruginosa*. This isolate co-producing GES-5 and the ESBL BEL-1 showed only decreased susceptibility to imipenem, meropenem, and ertapenem. A similar isolate carrying both enzymes, also showing only decreased susceptibility to all carbapenems, was recovered in 2009 from an ICU patient in Portugal (*23*).

We detected several OXA-181 producers that remained susceptible to imipenem and meropenem. This phenomenon has been reported previously (*37*) and has contributed substantially to the misrecognition of such carbapenemase producers and therefore to their silent spread. Nevertheless, we must underscore that all isolates from our collection could be detected by using available commercial selective media, such as the chromID CARBA SMART agar (bioMérieux).

The high rate (n = 12 [26%]) of carbapenem producers in our study being pandrug-resistant (i.e., having resistance to fluoroquinolones, aminoglycosides, tigecycline, and SXT) is of concern. The high rate of fluoroquinolone resistance was partly related to the occurrence of the *qnrS* gene. We have highlighted its endemic spread because it was identified in all of the carbapenemase-producing isolates identified, regardless of their clonal background and type of carbapenemase produced. Moreover, some isolates exhibited a worrying decreased susceptibility to ceftazidime/avibactam (MIC 3 mg/L; resistance breakpoint >8 mg/L), likely suggesting that some isolates actually started developing reduced susceptibility to that drug combination and moving toward resistance. Selection of KPC-producing *K. pneumoniae* exhibiting resistance to ceftazidime/avibactam has been observed recently in Italy, where KPC is endemic. This resistance occurred in particular through substitutions in the KPC enzyme, leading to increased hydrolysis of ceftazidime and decreased affinity to avibactam (*38*). In our study, we did not observe selection of KPC-3 variants; therefore, the reduced susceptibility probably is mainly related to the co-production of GES-5, permeability defects, or both. Nevertheless, all isolates remained susceptible to the combination ceftazidime/avibactam, independently of the clonal type and the carbapenemase produced (whether KPC-3, OXA-181, or GES-5). In addition, fosfomycin and polymyxins are still therapeutic alternatives because very few isolates exhibited resistance to these antimicrobial agents (3 to fosfomycin and 1 to polymyxins).

Our study has some limitations, given that we included isolates from a single hospital. However, this private hospital receives patients transferred from several public hospitals in Lisbon and its surroundings and therefore might reflect at least the global situation in this capital city of Portugal, which includes approximately one third of the national population. This phenomenon is evident in the diversity of enzymes, plasmids, and clonal backgrounds identified in our study.

In summary, KPC-3 was the most common carbapenemase identified in *K. pneumoniae* isolated at the SAMS Hospital in Lisbon, Portugal, followed by OXA-181, which emerged in 2016 and appears to be on the rise. In addition, a large proportion of isolates are pandrug-resistant, drastically diminishing the options for treatment. Finally, considering the increasing identification of carbapenemase-producing *K. pneumoniae* in this hospital, systematic carriage screening at hospital admission, additional surveillance studies, and early detection of such isolates are required to limit their further spread. These measures would help mitigate the spread of these isolates in Portugal and avert the endemic levels that have been observed in other countries in Europe.
